# Role of nanostructures in improvising oral medicine

**DOI:** 10.1016/j.toxrep.2019.04.004

**Published:** 2019-04-15

**Authors:** Shatadal Ghosh, Sumit Ghosh, Parames C. Sil

**Affiliations:** Division of Molecular Medicine, Bose Institute, P-1/12, CIT Scheme VII M, Calcutta, 700054, West Bengal, India

**Keywords:** AD, Alzheimer’s disease, AmB, amphotericin B, AMCNS, cationic niosome-based azithromycin delivery systems, AP, acetylpuerarin, AT1R, angiotensin II receptor type 1, BCRP, breast cancer resistance protein, CNL, conventional lipid nanoparticles, CSC, core shell corona nanolipoparticles, DCK, N-deoxycholyl-l-lysyl-methylester, DDS, drug delivery system, DM, diabetes mellitus, DOX, doxorubicin, EPR, enhanced permeability and retention effect, FRET, Foster resonance energy transfer, 5-FU, 5-fluorouracil, GI, gastrointestinal, GMO, glyceryl monoolein, IBD, inflammatory bowel disease, LG, Lakshadi Guggul, LNC, Lipid Nanocapsule, MFS, Miltefosine, MNBNC, Micronucleated Binucleated Cells, MSN, mesoporous silica nanoparticle, MTX, methotrexate, NP, nanoparticle, NPC, nanoparticulate carriers, NSAID, non-steroidal anti-inflammatory drug, OA, osteoarthritis, OXA, oxaliplatin, PAMAM, poly (amidoamine), PD, Parkinson’s disease, PEG, polyethylene glycol, PIP, 1-piperoylpiperidine, PLGA, polylactic-co-glycolic acid, PNL, PEGylated lipid nanoparticles, pSi, porous silicon, pSiO, porous silica oxide, PZQ, praziquantel, SLN, solid lipid nanoparticle, SMA, styrene maleic acid, SMEDD, self microemulsifying drug delivery system, TB, tuberculosis, Tmf, tamoxifen, TNBS, trinitrobenzenesulphonic acid, TPGS, tocopheryl polyethylene glycol succinate, WGA, wheat germ agglutinin, Oral medicine, Nanostructures, Drug delivery system

## Abstract

The most preferable mode of drugs administration is via the oral route but physiological barriers such as pH, enzymatic degradation etc. limit the absolute use of this route. Herein lies the importance of nanotechnology having a wide range of applications in the field of nano-medicine, particularly in drug delivery systems. The exclusive properties particularly small size and high surface area (which can be modified as required), exhibited by these nanoparticlesrender these structures more suitable for the purpose of drug delivery. Various nanostructures, like liposomes, dendrimers, mesoporous silica nanoparticles, etc. have been designed for the said purpose. These nanostructures have several advantages over traditional administration of medicine. Apart from overcoming the pharmacokinetic and pharmacodynamics limitations of many potential therapeutic molecules, they may also be useful for advanced drug delivery purposes like targeted drug delivery, controlled release, enhanced permeability and retention (EPR) effect. In this review, we attempt to describe an up-to-date knowledge on various strategically devised nanostructures to overcome the problems related to oral drug administration.

## Highlights

• Nanotechnology facilitates oral administration of drugs.
• Various nanostructures have been designed for this purpose.
• These nanoformulations are useful for many diseases.

## 1 **I**ntroduction

Different therapeutic agents face the problem of low oral bioavailability and the scientific solutions to these problems are highly challenging [1]. Oral administration of drugs is the most preferable route to deliver therapeutics because of the patient’s acquiescence and easy administration. Especially, it is the most desirable mode of drug administration for long term or daily use because of the convenience in doing so. This method is particularly essential when patients are admitted to the clinics in some developing countries when other modes of drug administration are limited [2]. In spite of the evident benefits of oral route of drug administration, a number of factors, like the external barriers in the gastrointestinal (GI) tract, makes it unsuitable for the purpose. Herein comes the role of smartly designed drug delivery systems (DDS) that can not only overcome the limitations of the barriers to oral administration but also enhance the efficacy of the treatment. DDS are major nanotechnological advancement in the field of nanomedicine [3,4]. They offer advantages of not only overcoming pharmacokinetic and pharmacodynamic limitations of bioactive molecules but also have added benefits like targeted delivery, controlled release under response of a stimulus like pH and so on [5]. Nonetheless, the harsh environment of the GI tract offers major challenges for designing of such smart DDS for oral administration as they will be subjected to breakdown and subsequent metabolism. But a well thought and tailored designed DDS can partially or fully protect the cargo from the harsh environmental degradation in the stomach and the GI tract due to its encapsulation into the nanostructures that act as their carrier. These difficulties add to the poor functioning of nanostructures via oral administration [6]. Therefore, a careful and intelligent designing system is needed. However, an ample room for improvement is available and therefore, optimization of a number of factors is required for development of an intelligently designed DDS that can overcome the above mentioned limitations.

This review accounts briefly about the nanostructures developed drug delivery so far, for oral administration. Also, it describes the advantages and limitations of using such nanostructures for various drugs or natural products having beneficial effects on health and different transport mechanisms across the barriers of the GI tract. Most importantly, a detailed and up to date discussion of the usefulness of those nanostructures for oral medication against some diseases has been explored here.

## 2 Oral administration and nanostructures

The oral mode of administration has several advantages like easy administration, being painless, minimal chances of elicitation of immune system, easier uptake and wide assimilation/distribution of the drug [7]. Nanostructures used for drug delivery have been designed to accomplish targeted drug delivery, overcoming the pharmacokinetic and pharmacodynamic limitations of potential therapeutic molecules, optimizing the dose of the drug with narrow therapeutic window and reducing the side effects [5]. Although, the scope of nanostructures seem promising but they have few disadvantages too. Sometimes, nanostructures may elicit immune response. Moreover, the first pass effect makes the nanostructures less effective, as most of them get trapped in the liver or the spleen due to intra peritoneal administration. So, intelligently designed nanostructures and their oral administration may pave way for development of effective therapeutics for better treatment of diseases [[8], [9], [10], [11], [12], [13]].

## 3 The barriers of the GI tract

**Fig. 1:**
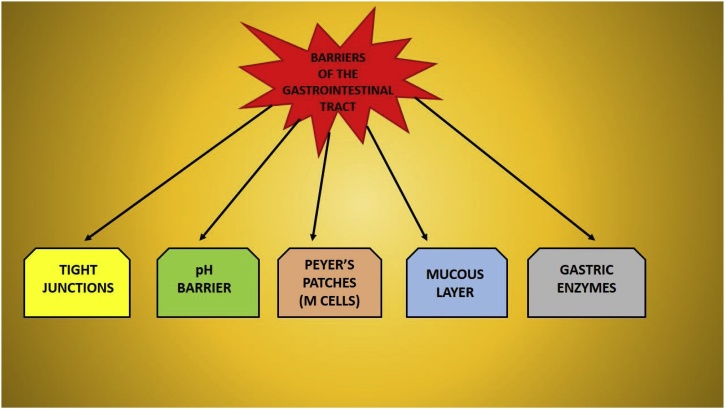
Barriers of the Human Gastrointestinal Tract.

The major barriers of the GI tract that offer challenges for designing a nanostructure for drug delivery are the pH of stomach, the mucus layer of the gastrointestinal epithelial liningand the tight intracellular junctions (Fig. 1).

The first barrier of the GI tract is the varying pH of the GI which varies from the acidic range in the stomach and rises to alkaline in the small intestine and then slightly falls in the caecum only to rise again in the left colon [14]. This fluctuation in the pH of the GI tract is essential for activation of various digestive enzymes and food absorption. But these abrupt changes in the pH of the GI tract, offers a major challenge for the development of an effective nanostructure.

Another major challenge is the thick mucus lining of the GI tract which serves as a protective barrier [15]. The nanostructures get trapped in the lining system and are cleared from the system without being absorbed [7].

Besides, bile salts exert their effects on the structure and function of drugs. The absorbed drugs are then subjected to the “first-pass metabolism” in liver before reaching the systemic circulation leading to further degradation [16,17]. Overall, these major barriers need to be overcome for devising an efficient DDS for oral route of administration.

## 4 Nanostructures for drug delivery by oral administration

### 4.1 Liposomes and lipidic nanostructures

**Fig. 2:**
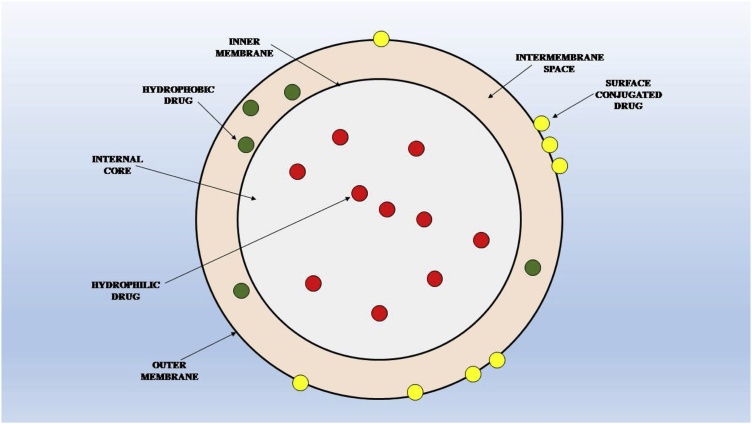
Membrane permeability of different types of drugs.

The liposomes have the composition quite similar to the cell membrane. These entities entrap the particular drug and can easily fuse with the cellular membranes for drug delivery (Fig. 2). The excellent biocompatibility of the liposomal formulations makes them highly desirable for the purpose of drug delivery. Liposomal formulations are the most widely used vehicles for lipophilic drug delivery via the oral mode [18]. The liposomal formulations facilitate the absorption and transepithelial transport of the lipophilic drugs mainly by three mechanisms: they can increase the dissolution of the drug in the intestinal environment, enhance the drug transport through interaction with the enterocytes and reduction of the first pass effect. The size and composition of the liposomes are important determinants for successful drug delivery [19]. The most important determinant of successfully delivering a drug via oral mode is the fate of the vehicle in the intestinal environment. The two major threats to liposomal formulations are their degradation and entrapment in the mucinous layers lining the GI tract. The vehicles should be designed as such they are acid resistant and are not a substrate for the gastric enzymes. This prevents the absorption and transport of the liposomal formulations resulting in the excretion of the vehicles and low bioavailability [20]. A major advantage of liposomal formulations is that, when up taken by the intestinal cells, they are absorbed and transported by the lymphatic system [21]. These result in minimizing the first pass effect of the liposomal formulations resulting in the elevated concentration of the administered drug in the systemic blood, increased bioavailability and site targeting [22].

**Fig. 3:**
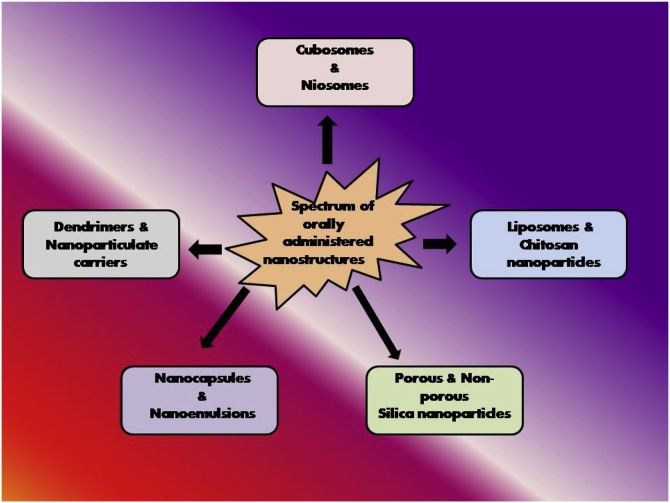
Different types of nanomedicines which can be orally administered.

**Fig. 4:**
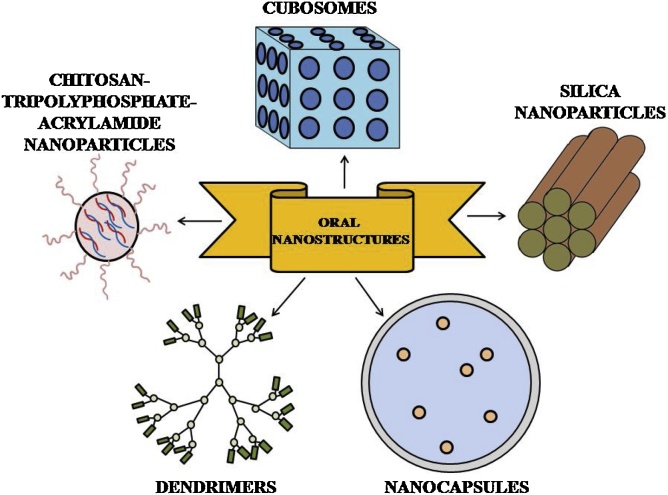
Diagrammatic representation of various oral nanostructures.

A number of novel drug delivery systems have been developed to elevate the bioavailability of lipophilic drugs via oral mode of administration including solid dispersions, nanocrytals, cyclodextrin complexes and lipid nanocarriers such as micelles, lipid nanoparticles (NPs) and nanoemulsions (Fig. 3, Fig. 4) [23]. Of the various modifications, PEGylation of vehicles provides excellent increase in biocompatibility and thereby the bioavailability of the vehicles. A significant increase in the permeability and absorption of paclitaxel is reported when delivered via PEGylated polyanhydride [24]. The bioavailability of acetylpuerarin (AP) was enhanced by oral administration of D-α-tocopheryl polyethylene glycol succinate (TPGS) stabilized nanoemulsions, in a focal cerebral ischemia-reperfusion mice model to study brain injury [25]. Zhang et al showed that PEGylated Lipid nanoparticles (PNLs) has the most sustained and delayed drug release than Conventional Lipid Nanoparticles (CNLs), which also increased absorption and reduced lipolysis [23]. Conventional Solid Lipid Nanoparticles (SLNs) were trapped by highly viscoelastic mucus. Increased permeability of PEGylated SLNs has been reported in mucus secreting HT29/Caco2 co-culture monolayer [26]. Bile salt liposomes have been found to enhance lymphatic transport and oral bioavailability of paclitaxel [27]. Freeze dried probilosomes enhance oral administration of cyclosporine A, thereby acting as potential nanocarriers [28]. Histidine tagged EphA2 receptor specific peptide anchored docetaxel liposomes act as oral agents for the healing of lung cancer [29]. Oral administration of ginger based nanolipids loaded with siRNA have been reported to function as efficient siRNA drug delivery system for the treatment of colitis [30]. Oral bioavailability of notoginsenoside R1 has been found to get improved with sodium glycocholate-mediated liposomes [31]. Chitosan derivative coated liposomes have been found to induce sustained release and improve oral bioavailability of curcumin [32]. Chitosan-thioglycolic acid-pluronic F127 (CS-TGA-PF) liposomes with enhanced mucus adhesive and penetrating ability have been found to enhance oral delivery of paclitaxel [33].

The development of Nanostructured Lipid Carriers (NLCs) as novel carriers was reported recently. NLCs overcame the limitations of SLNs and also improved the pharmacokinetics of triptolide (TP) [34]. They also pointed that the NLCs are better than polylactic-co-glycolic acid (PLGA) because of two properties. First, they can adhere to the gut wall due to high surface area of the ultrafine dispersion. Secondly, the metabolised secondary derivatives of the parent vehicles further increases the dissolution as well as the absorption of the drugs, which is further facilitated due to presence of emulsifying agents like bile salts. Further, as mentioned before, the liposomal preparations seem to escape the first pass effect due to absorption and transport by lymphatic system. Tacrolimus loaded lipid nanocarriers have been found to enhance in vivo bioavailability when administered through the oral route [35]. N-acetyl-L-cysteine functionalized lipid nanocarriers have been found to improve oral bioavailability of curcumin [36]. Alginate based lipid nanocarriers have been used successfully for the administration of amphotericin B via the oral route [37].

### 4.2 Niosomes

Niosomes are non-lecithoid carriers, which have structural similarities with liposomes. They are vesicular systems synthesized from non-ionic surfactants. They are biodegradable and biocompatible and can carry both lipophilic and hydrophilic drugs. They were designed to overcome the limitations of liposomes, particularly those related to phospholipid oxidation. Cationic niosome-based azithromycin delivery systems (AMCNS) were successfully developed by Zhong et al. [38].

Glimepiride and nateglinide loaded niosomes have been reported to increase the oral bioavailability in comparison to the free drug [39,40]. Oral administration of Telmisartan loaded niosomes have been found to significantly reduce the blood pressure in methyl prednisolone acetate mediated hypertensive Wistar rat models and also attenuates the expression level of angiotensin II receptor type 1 (AT1R) gene [41]. Studies on oral administration of Tramadol HCl encapsulated niosomes to mice were found to exhibit significant analgesic effects compared to the Tramadol HCl solution [42]. Niosomes can carry multiple cargos including drug, gene, vaccine, etc. and has been reported to be administered via multiple modes like topical, parenteral and oral routes. The versatility of niosomes makes it an attractive agent for oral nanostructure.

### 4.3 Silica nanoparticles (SiNPs)

In the complicated environment of the GI tract, SiNPs can offer major advantages for drug delivery purposes. Along with protecting the hydrophobic drug from the harsh intestinal milieu, the SiNPs are resistant to low pH. Thus, the SiNPs offer clear advantages for oral drug delivery purpose.

Apart from non-porous SiNPs, the porous SiNPs has drawn much attention due to properties like pore-size tunability, high pore volume, ordered pore structure, high surface area for functionalisation, high thermal stability and low-pH resistance. Mesoporous SiNPs (MSNs) have size ranging from 100 to 200 nm. This leads to high amount of drug loading into the mesoporous particles and provide protection to the water insoluble drug from the harsh microenvironment and sustained release at the targeted delivery site. With the appropriate surface modification options available, the mesoporous SiNPs can be modified in a way to make it more water soluble. The most common surface modification is PEGylation, for increasing the water dissolution and increased biocompatibility. The large surface area and pore volume of water insoluble porous silicon (pSi) and porous silica oxide (pSiO) particles improves adsorption of drugs and also allow the drug molecules to remain dispersed within their pores. Oral absorption of molecules/drugs, such as antipyrine, ibuprofen, naphthalene, ranitidine, furosemide, indomethacin, insulin, telmisartan, itraconazole and vancomycin loaded into mesoporous pSi and pSiO particles have already been investigated [[43], [44], [45], [46]].

Amine functionalized cubic mesoporous silica nanoparticles act as efficient oral delivery systems for the enhancement of bioavailability of curcumin [47]. Oral administration of poly-ethylene glycol functionalized mesoporous silica nanoparticles have been found to targetfully modulate differentiation of cells of distinct regions of the gastrointestinal tract [48]. Clofazimine encapsulated silica nanoparticles have been used for the oral treatment of antibiotic resistant *Mycobacterium tuberculosis* infections [49].

In spite of the unique properties of silica, their use for drug delivery is limited as detailed in-vitro and in-vivo studies need to be carried out before drawing a final conclusion about the toxicity profile of the silica NPs.

### 4.4 Dendrimers

Dendrimers are polymeric nanoparticles, chemically synthesized to possess shape, size and nanoscopic physicochemical properties similar to those of the proteins. They are almost spherical shape with branched entities having diameters 2 and 10 nm. Poly(amidoamine) (PAMAM) dendrimers have been extensively studied in anti-inflammatory, anticancer, antimicrobial, antiviral and other types of drug formulations. PAMAM dendrimers are biocompatible, non-immunogenic, water-soluble and have amino and amide functional groups that can be modified for enabling connection with target molecules. Several varieties of dendrimers are available for oral administration, like G3 PAMAM loaded with propranolol, G5 PAMAM loaded with ketoprofen, G0 PAMAM loaded with furosemide, etc [50]. Recently, oral administration of albendazole via the lyophilized mucoadhesive dendrimer enclosed matrix tablets improves its half-life and pharmacokinetic profile [51].

### 4.5 Cubosomes

Cubosomes are liquid crystalline nanoparticles of certain surfactant having a specific proportion of water blended with its microstructure which contributes to its exclusive properties [52]. The most common surfactant used for making cubosomes is the monoglyceride glycerol monoolein. The oral administration of amphotericin B (AmB) suffers from the problem of meagre bioavailability. Studies have shown that GMO cubosomes improve the efficiency of AmB via the oral route [53]. On the other hand, monoolein cubosomes enhance the oral bioavailability and solubility of poorly water-soluble drugs [54]. Recent studies have been shown that cubosomes act as efficient oral drug delivery systems in enhancing the release of clopidogrel bisulphate in the intestine [55].

## 5 Oral nanostructures for vaccination

The best prophylactic approach to combat infectious disease is vaccination, both because of its efficacy and cost-effectiveness. Various agents like weakened or killed pathogens, pathogenic proteins or peptides can elicit the desired immune response. Oral vaccine delivery is advantageous because it confirms mass immunization, lucid protocol for administration, easy storage, low production cost and reduced chances of infection. Moreover, oral vaccines function as potential injectable vaccines, producing antigenic specific antibodies in the blood and mucosa. Unlike injectable vaccines, oral vaccines do not need a high percentage of purity. The oral route is characterized by numerous obstacles with respect to the effective delivery to the immune cells. Oral vaccines suffer from exposure to proteolytic enzymes, acidic pH and bile salts which lead to their degradation in the GI tract. Vaccines must also overcome various biological barriers of the GI tract. Thus, for sustained immunogenic effect, oral vaccines require several doses of administration than their systemic counterparts [56].

An effective oral drug delivery agent should be able to survive the harsh condition of the GI tract. Also, it should not elicit any allergic response. Nanoparticles are gaining popularity in this field also because of their property of co-delivery of antigens and adjuvants. Multilayer engineered nanoliposomes have also been successfully used for the oral administration of lipopeptide based vaccines against group A streptococcal infection [57]. Recently, oral vaccination with Omp31 loaded *N*-trimethyl chitosan nanoparticles has been found to induce immunity against *Brucella melitensis* [56]. Oral administration of acid resistant *Helicobacter pylori* vaccine encapsulated (HP55/PLGA) nanoparticles has been reported to promote immune protection [58].

## 6 Nanoparticles for oral protein delivery

Oral protein delivery is a biotechnological advancement that has made a considerable progress in recent period. Although the oral route of administration is the most favoured one but poor bioavailability has been observed for the orally administered protein at different therapeutic doses. The reasons include high molecular weight, poor membrane permeability and degradation of the protein in the intestinal milieu. However, the nanoparticles put hope serving as carriers for the oral delivery of the proteins [59]. The nanoparticles, not only protect these proteins from the harsh intestinal microenvironment (from the varying pH range and digestive enzymes) and facilitate the uptake and transport of them across the gastrointestinal barriers but also improve their bioavailability [60]. An applicative example is that the risks of intravenous or subcutaneous administration of insulin can be overcome by the oral delivery of insulin via the mesoporous silica nanoparticles (MSNs) owing to their controllable morphology and high loading efficiency [58].

Receptor specific ligand enhances the chances of uptake by the epithelia and thus increases absorption. Several ligands have their own specific mode of transport; like glucose coated nanoparticles are uptaken by caveolin-mediated pathways whereas insulin modified nanoparticles are uptaken by clathrin mediated pathways. Also, ligands uptaken by specific cells can further aid in tissue targeting. In addition, modifications of nanoparticles using targeting peptide have been shown to enhance manifolds bioavailabilty of the drugs [14].

## 7 Application spectrum of oral nanostructures for treatment of various diseases

### 7.1 Alzheimer’s disease

Alzheimer’s disease (AD) is a common form of dementia, a memory and personality disorder, which lacks any available effective therapeutic treatment till date [61]. The disease progresses with the accumulation of amyloid-β peptide and intracellular neurofibrillary tangles of the tau protein in its hyperphosphorylated form [62]. In 2010, about 35 million people worldwide were afflicted with AD. The inception of disease symptoms usually begins in people who are above 65 years old [63,64].

In the quest of reducing side effects, the use of herbal medicines for the treatment of AD has been increased in the last few years. Several medicinal plants possessing significant beneficial effects on the central nervous system (CNS) and antioxidant properties have been found to improve the cognitive functions in AD patients. However, the therapeutic effects of such orally administered medicinal extracts are hampered by different barriers. For example, the effects of piperine (PIP) [a nitrogenous alkaloid, obtained from the fruits of long pepper (*Piper longum*), black pepper (*Piper nigrum*), and other species of the Piperaceae family] on memory and neurodegeneration in animal model of AD has been reported but the oral delivery of PIP has been found to suffer from its hydrophobicity and presystemic metabolism [65]. Glyceryl monooleate has a self-assembling property which helps in maintaining lower surfactant concentration in dispersion systems and thus acts as an effective nanocarrier for oral delivery. Recently, characterization of novel Tween-modified monoolein cubosomes (T-cubs) based on polydispersity index, particle size, zeta potential, in vitro release, has revealed their potential as nanocarriers for brain-targeted oral delivery of PIP. Studies have shown that the T-cubs can significantly sustain in vitro release and enhance cognitive effects of PIP. They can even restore cognitive functions to their normal level. The T-cubs have been found to enhance the anti-apoptotic and anti-inflammatory activity of the loaded PIP thereby indicating their potential role in stopping progression of the disease [66].

Liposomes and chitosan nanoparticles have also been found to be effective for brain targeted oral delivery of curcumin derivatives and tacrine respectively [67,68]. Curcumin, a diarylheptanoid obtained from the ginger family Zingiberaceae, decreases amyloid-β plaques, neuronal degeneration and metal chelation, thereby helping in improving cognitive functions in AD patients [69]. Tacrine, on the other hand, is a potent cholinesterase inhibitor, which helps in improving cerebral blood flow and plaque depletion in AD patients [70].

Yusuf et al. have reported that P-80 coat imparts brain specific targeting to solid-lipid nanoparticles containing piperine. These nanoparticles by virtue of their aptitude in crossing the blood-brain barrier are considered to be effective in exerting therapeutic effects in AD model. Studies have also shown that oral administration of P-80 coated PLGA estradiol loaded nanoparticles results in significantly elevated levels of the hormone in the brain within 24 h as compared with uncoated ones in murine model of AD [71].

Pegylated biodegradable dexibuprofen nanospheres administration to APPswe/PS1dE9 mice easily crosses the blood brain barrier without disrupting it and decrease brain inflammation through reduction of β-amyloid plaques [72].

**Fig. 5:**
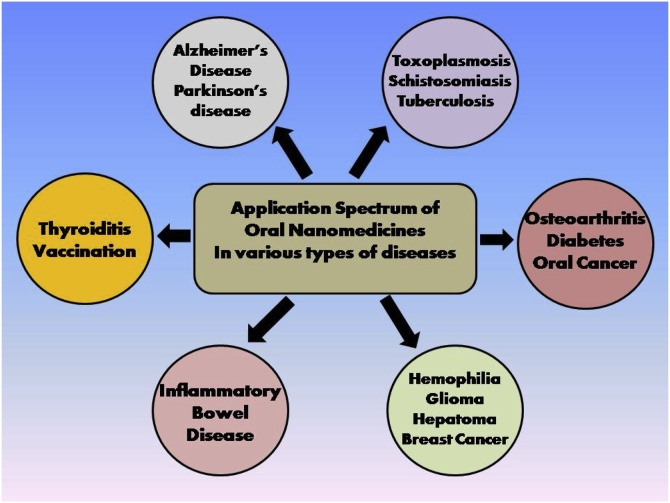
Application of drug loaded nanostructures for the treatment of different types of diseases.

Thus, oral nanomedicine is a novel approach in the treatment of Alzheimer’s disease (Fig. 5).

### 7.2 Schistosomiasis

Schistosomiasis is a parasitic disease, the causative agent of which are trematodes of the genus Schistosoma. Early symptoms of the infection include fever, severe abdominal pain and blood in stools or urine. Schistosomiasis affects more than 210 million people worldwide and almost 200,000 people die from it every year. The disease is common in Asia, Africa and South America [73].

Miltefosine (MFS), an alkyl phosphocholine has been reported to exert therapeutic effects against various developmental stages of *Schistosoma mansoni*. Thus, MFS has been proposed as a potent anti-schistosomal drug. The Lipid Nanocapsule (LNC) has a hybrid structure between nanocapsule polymers and liposomes because of its oily core which is surrounded by a tensioactive membrane, thereby conferring great stability to the structure. Studies have shown that MFS-LNCs (MFS-Lipid Nanocapsule) can be considered as novel oral nanovectors as they have the combined bioactivity and pharmaceutical advantages of both MFS and LNCs respectively. Thus, MFS-LNCs can be considered as novel oral nanomedicines for the therapy of schistosomiasis [74].

De Souza et al. developed and investigated the biological applications of PZQ loaded solid lipid nanoparticles (SLN). Cytotoxicity studies showed that encapsulation of PZQ into SLN reduced the toxicity in HepG2 cells compared to free PZQ. In culture of *Schistosoma mansoni*, results showed that PZQ loaded SLN were more efficient than free PZQ in a concentration dependent manner. Thus, PZQ loaded SLN could be a new drug delivery system for the treatment of schistosomiasis [75].

Praziquantel loaded lipid nanocapsules act as potential nanotherapeutic agents against *Schistosoma mansoni* by enhancing the reduction of worm burden, amelioration of hepatic pathological changes and damaging the fluke suckers and tegument when administered orally in rats [76]. On the other hand, self nanoemulsifying drug delivery systems (SNEDDs) have been found to augment anti-schistosomal activity of epiisopiloturine when administered via the oral route [77].

### 7.3 Osteoarthritis

Osteoarthritis (OA) is a type of joint disease that results from gradual erosion of joint cartilage and breakdown of the underlying bone. The most common symptoms include severe joint pain and joint swelling. OA is the most common form of arthritis affecting about 3.8% of the world population as of 2010. It is commonly observed in men before 45 years of age, while after 45 years of age, it is more common among women. It becomes more common in both sexes with the increase of age [78].

Studies have shown that iron saturated bovine lactoferrin loaded in alginate-chitosan polymeric nanocarriers (AEC-CP-FebLf-NCs) diminish IL-1β induced oxidative stress and chondrocyte proliferation in OA induced murine model. Oral administration of such nanocarriers reduce joint inflammation and downregulates the expression of NO, IL-1β, JNK and MAPK. It also up-regulates calcium metabolism, type II collagen and inflammation depleted iron via inhibition of iron transporting receptor miRNA and leads to the dissolution of calcium pyrophosphate crystals found in the joints of the experimental animals [79]. The same group conducted another novel experiment to determine the anti-arthritic efficacy of Lakshadi Guggul (LG) and *Cissus quadrangularis* encapsulated in alginate-enclosed chitosan-calcium phosphate nanocarriers (NCs). They have conducted experiments both *in vitro* in primary human chondrocytes and *in vivo* in collagen-induced arthritic mice model. Results indicated that LG increase viability and inhibited mitochondrial depolarization and consequent cellular apoptosis. *in vivo* studies also revealed cartilage regenerative activity [80].

A high dose of ibuprofen (2400 mg/day), a non-steroidal anti-inflammatory drug (NSAID), used for the treatment of osteoarthritis, is associated with a wide range of side-effects. Oral controlled release formations of ibuprofen polymerized in conjugation with PLGA has been found to effectively control the colonic release and absorption of the drug thereby increasing its efficacy by minimizing the associated side-effects [81].

Thus, oral nanostructures may also be helpful for treatment of arthritis patients as well.

### 7.4 Inflammatory bowel disease

Inflammatory Bowel Disease (IBD) is an autoimmune inflammatory disorder affecting various parts of the colon and intestine. It is associated with the T_H_1, T_H_2 and T_H17_ pathways of immune response. The disorder encompasses two major types of diseases- Crohn’s disease and Ulcerative colitis. Increased evidence of IBD, after World War II, was found to be correlated with increased meat consumption. This pointed towards a possible role of animal proteins in induction of the disease [82].

Treatment of IBD demands improvement of colon targeted drug delivery systems. Alteration of GI tract physiology and induction of inflammation is a major problem for drugs targeted to the colon. However, oral nanomedicines have helped in enhanced uptake of such drugs in the diseased section of colons bypassing the aforesaid problems [83].

Physiological changes in the intestine through alteration of pH, nutrient absorption, digestion and egestion reduce the bioavailability of colon targeted drugs [84,85]. Therefore, several nanostructures have been designed to increase the efficacy of orally administered colon targeted drugs. Size, surface charge (mucoadhesives and bioadhesives), PEGylation, pH dependent as well as biodegradable and redox oral nano-delivery systems have been reported to augment colon targeted drug delivery in IBD patients [83]. Nanoparticles on entering the GI tract undergo internalization via endocytosis, into the epithelial cells of the GI tract. In case of IBD, specialized epithelial M cells uptake nanoparticles through transcytosis. Translocation of nanoparticles can also occur by persorption through the gaps in the villous tips [86]. Therefore, size of the nanoparticles is crucial in determining their efficacy as drug delivery systems. Surface charge is another important property which determines the deposition pattern and therapeutic efficiency of oral nanoparticles. Cationic nanodelivery systems adhere to the mucosal surface of the inflamed tissue through interaction of negatively charged intestinal mucosa with the positively charged nanocarrier [87]. Anionic nano-delivery systems get adhered to the inflamed tissue through the electrostatic interaction with elevated concentration of positively charged proteins in inflamed portions [88]. On the other hand, PEGylation of nanostructures prevent their interaction with mucous constituents and helps in translocation into the inflamed regions of the intestinal epithelium thereby increasing the reliability of the drug delivery system as a whole [89]. The pH level of the small intestine is usually higher than the remaining portion of the GI tract. Therefore, coating of oral nanoparticles carrying the concerned drugs with pH sensitive biocompatible polymers increases the therapeutic efficiency of the system as a whole [90].

It has also been observed that the anti-inflammatory and anti-diarrheal effects of vasoactive intestinal peptide can be maximized when administered as an oral nanomedicine in the form of conjugation of sterically stabilized micelles and thus act as a potential therapeutic tool for the treatment of ulcerative colitis [91].

Overexpression of CD 98, a heterodimeric type II membrane glycoprotein in the inflamed intestinal tissue has been successfully targeted with orally administered hydrogels bearing CD 98 siRNA loaded nanoparticles in murine model of DSS induced colitis [92].

Thus, oral nanomedicine has provided breakthrough solutions to problems related to colon targeted drug delivery systems.

### 7.5 Parkinson’s disease

Parkinson's disease is a neurodegenerative disorder of the central nervous system which mainly affects the motor system [93]. The motor symptoms of Parkinson's disease result from the death of dopamine generating cells of the substantia nigra of the midbrain. The visual symptoms of the disease include muscular rigidity, gait, slow movement, difficulty in walking, thinking and behavioural problems, sleep, dementia, depression and emotional problems. It commonly occurs in people who are above 50 years old [94]. Resveratrol is a potent antioxidant and is reported to exhibit therapeutic effects in PD patients. However, its oral bioavailability is hampered due to hepatic and first pass circulation. To combat such problems, vitamin E loaded resveratrol nanoemulsions have been formulated. The nanoemulsions are composed of vitamin E:sefsol (1:1) as the oil phase with Tween 80 as the surfactant and Transcutol P as the co-surfactant. Oral administration of such brain targeted nanoemulsions has been found to successfully reduce oxidative stress, thereby paving a novel path for treatment of the disease [95]. It has been reported that oral administration of a nanocrystal formulation of Schisantherin A, a Chinese herbal agent, improves bioavailability and delivery to the brain in Parkinson model. Its neuroprotective effect is mediated by the activation of the protein kinase B (Akt)/glycogen synthase kinase-3β (Gsk3β) pathway [[96], [97], [98]].

### 7.6 Tuberculosis

Tuberculosis (TB) is an infectious disease caused by different strains of *Mycobacterium tuberculosis* usually affects the lungs, but can also affect other organs of the body. It spreads through air by coughing and sneezing of the infected patients. In 2013, 9 million cases of infection had been registered including 1.3 million associated deaths in developing countries. Oral route of drug administration is a common therapeutic approach for tuberculosis. However, high dosage frequency of these drugs is the cause for side effects. Studies have shown that Wheat germ agglutinin (WGA) functionalized poly (lactide-co-glycolide) nanoparticles (PLG-NP) can act as potential carriers for such drugs through the oral route. Such nanomedicines help in reducing dosage frequency of anti-tubercular drugs, thereby increasing the credibility of TB chemotherapy [99]. Studies related to oral administration of PLG nanoparticle encapsulated anti-tuberculosis drugs (isoniazid + pyrazinamide + rifampicin + ethambutol) has also shown convincing results for cerebral drug delivery in murine model [100].

Another study showed the development of PLGA-based nanoformulation. Levofloxacin incorporated in this system of tuberculosis showed a sustained release in plasma and considerable efficacy against multidrug resistant tuberculosis. Moreover, this particular formulation did not show any significant adverse effects in experimental mice [101].

There have also been reports of antituberculosis drugs like moxifloxacin and rifampicin being able to trigger an immune response in lung macrophages when administered after loading into gelatin and polyisobutyl cyanoacrylate nanoparticles [102].

Clofazimine encapsulation in nanoporous silica particles have been successfully administered orally for the treatment of antibiotic resistant TB infections [103].

Matryoshka type gastro-resistant microparticles containing rifampicin loaded PLGA nanoparticles bears a methacrylic acid-ethyl acrylate based coating to protect the loaded antibiotic from degradation under various gastric conditions. The inner core bearing the antibiotic is released under specific intestinal conditions whereas the outer coating protects them from degradation. Oral administration of this drug delivery system ensures the sustained release of rifampicin and subsequent effect against *Mycobacterium tuberculosis* [104].

### 7.7 Diabetes mellitus

Diabetes mellitus (DM) is a chronic disease associated with the incapability of the β cells of pancreas to produce sufficient insulin or ineffective use of insulin to control glucose homeostasis [105]. DM is of mainly two types: Type 1 diabetes (DM1) and Type 2 diabetes (DM2). Currently, almost 347 million people worldwide have diabetes, most of them (˜ 90%) having the symptoms of DM2 [106]. Treatment of DM1 mainly comprises of insulin administration whereas that of DM2 is generally more complex. DM2 patients are primarily advised to adapt to a lifestyle of controlled carbohydrate intake and elevated physical exercise [105]. If this new lifestyle becomes insufficient to control glycemia, therapeutic intervention is essential. The preferred medication involves the use of oral hypoglycaemiants and insulin secretagogues. Recently, combinations of different drugs have gained importance to treat DM. For instance, the combination of insulin sensitizers and insulin secretagogues has been proven to be effective in targeting both the increased levels of circulating insulin as well as augmented hormonal efficacy [105]. Though DM2 is caused by the insufficient insulin response in different tissues and organs, it is primarily associated with decreasing beta-cell function and subsequent poor insulin secretion. Naturally, DM2 patients very often need exogenous insulin to maintain glucose homeostasis [107]. Incretin based therapy uses incretin hormones glucose-dependent insulinotropic peptides and glucagon like peptide-1 for the treatments of the Type II DM. Nanosystems like mesoporous materials have been devised to overcome the limitations of various incretin hormone formulations [108].

The most common route for exogenous insulin delivery is subcutaneous administration. Numerous formulations for subcutaneous insulin delivery have also been developed. Though all of these insulin formulations are effective for managing glycemia, all of them have the same disadvantage of subcutaneous administration. And this is not the only reason why scientists try to find out alternative modes of insulin administration. Insulin does a lot more job than just controlling plasma glucose levels in the body [109]. Insulin is secreted by beta-cells of pancreas and released directly into the portal vein. Therefore, beyond the pancreas, the insulin helps in blocking glucagon secretion from the liver. Therefore, the liver becomes the major target for insulin and a major site for insulin clearance. This hepatic insulin controls a number of physiological processes such as liver gluconeogenesis, glycogen storage and production, lipid metabolism and homeostasis, proteolysis and hepatic regeneration [107]. But, subcutaneously administered insulin, though efficient in controlling glycemia, fails to exert the above mentioned hepatic actions. Actually, insulin secreted from pancreas creates a concentration gradient extending from the pancreas to the liver, and beyond to other organs and tissues. However, this gradient is formed in the opposite direction in case of subcutaneously administered insulin. Recent research reveals that, this gradient is important for other broader actions of insulin, including lipid and amino acid homeostasis, hepatic tissue regeneration, etc. Different technologies, like insulin pump and inhalable insulin, have been adopted to overcome this problem. However, these strategies have severe limitations as they are also unable to produce the insulin gradient between the pancreas and liver. Moreover, promise of inhalable insulin treatment was further cut short for much higher costs [110].

Oral administration of insulin requires the production of nanocrystals from this peptide hormone and subsequent incorporation into nanostructures that ensure their stability and uptake. Administration of active, stable insulin into the system becomes even more challenging as it induces aggregation after solubilisation in non-nanostructured formulations. These difficulties further make the way for the development of oral administration of insulin by the help of nanosystems. In a study, oral absorption of insulin was improved by the use of a novel carrier of Vitamin B12 (Vit B12) gel core solid lipid nanopaticles (Gel-Core-SLN, GCSLN). Sol-gel conversion after ultrasonic heating and the use of double emulsion technology were applied to incorporate the insulin containing gel into the solid lipid nanoparticles (SLN). In vivo studies exhibited elevated absorption of insulin with a relative pharmacological availability (PA) of 9.31% compared to the normal insulin loaded SLN and GCSLN and fairly stable blood glucose levels up to 12 h were maintained without any spiky fluctuations. The study suggests Vit B12-GCSLN containing insulin as a potent nanocarrier for oral delivery of biomacromolecules with relatively elevated pharmacological availability [111].

One of the recent studies reported the interaction between insulin and silica nanopaticles (SiNP) funtionalised with mucoadhesive polymers (sodium alginate, chitosan or polyethylene glycol). Mucoadhesive polymers were used to facilitate high contact between the gut mucosa and the nanostructures to enhance the oral insulin bioavailability. This study showed high biocompatibility (at the tested concentrations of 50–500 μg/mL) of those nanostructures in Caco-2 and HepG2 cell lines as they mimic in vivo the target of nanoparticles loaded with insulin upon oral administration [112].

Another study demonstrates the production and characterization of PEGylated silica nanoparticles (SNP-PEG) for the oral administration of insulin. Depending on the molecular weight of PEG, the researchers have used two types of PEG like PEG 20,000 and PEG 6000. Kinetics study also revealed that drug release for uncoated and SiNP-PEG followed second order kinetics at pH 2.0. But at pH 6.8, that of SiNP-PEG followed first order kinetics and SiNP showed Boltzmann behaviour [113].

Zinc oxide, cerium oxide and silver nanoparticles have also been reported to exhibit anti-diabetic properties when orally administered in streptozotocin-induced diabetic Wistar rats [114]. On the other hand, eprosartan mesylate loaded nanobilosomes comprising of varying ratios of soybean phosphatidylcholine or sodium deoxycholate when administered orally is found to decrease the expression of inducible nitric oxide synthase (iNOS), angiotensin II type 1 receptor and transforming growth factor-β1 (TGF- β1) in Wistar rats [115].

### 7.8 Toxoplasmosis

Toxoplasmosis is caused by *Toxoplasma gondii*. The symptoms include muscle aches, eye problems, seizures and poor coordination. When infected during pregnancy, congenital toxoplasmosis occurs and usually affects the child. It spreads through exposure to infected cat faeces, the ingestion of poorly cooked food containing cysts and during pregnancy if the mother is infected. Rarely the disease spreads through blood transfusion. The parasite reproduces sexually in the cat family. However, it can infect all types of warm-blooded animals. About half of the world's population is infected with toxoplasmosis. Oral administration of Triclosan (TS) loaded liposomes against the virulent strain of *Toxoplasma gondii* (*T. gondii*) in murine model has been found to induce significant reduction in mortality rate and infectivity of tachyzoites that had been harvested from infected mice. Liposomal formulations of TS enhance its efficacy and allow its use at a lower dose [116].

Owing to the anti-microbial activity of lactoferrin, recent studies on the therapeutic effects of orally administered alginate chitosan calcium phosphate bovine lactoferrin nanocapsules in infected BALB/c mice have revealed that it results in elevated levels of reactive oxygen species, nitric oxide and Th1 cytokines which trigger parasitic clearance [117].

It has also been observed that oral administration of spiramycin-loaded chitosan nanoparticles (SLCNs) in infected Swiss albino mice improved the histopathological features of the brain, spleen, liver and eye thereby confirming its anti-parasitic effects [118].

Curcumin loaded nanoemulsions have been reported to treat acute and chronic toxoplasmosis, especially the latent bradyzoites in brain in mice model [119].

## 8 Other applications of oral nanomedicine

Curcumin is an important anticancer agent. It targets and silences a wide array of tumor growth related enzymes in case of glioma, hepatoma, breast cancer, colorectal cancer, etc. Oral nanoparticle formulations enhance curcumin dispersion in aqueous phase. The various modes of curcumin nanoformulations include polymeric nanoparticles (20–200 nm), micelles (5–10 nm), liposomes (50–150 nm), solid lipid nanoparticles (100–150 nm), nanogels (70–100 nm) and cyclodextrin inclusions. The curcumin nanoparticles conjugated with protein ligands and antibodies enable them to be tracked in the bloodstream. Such nanoparticle formulations increase the therapeutic efficacy of curcumin [120].

Combination therapy involving chemotherapy coupled with nanoparticle mediated drug is a novel approach in the field of cancer therapy. Studies have shown that orally administered doxorubicin-methotrexate loaded nanoparticles (DOX-MTX NPs) prevent progression of oral cancer in a 4-nitroquinoline-1-oxide induced oral squamous cell carcinoma (OSCC) model of rat. DOX-MTX NP decreases the mRNA levels of MMP-2 (Matrix metalloproteinase-2) thereby preventing tumor invasion and metastasis [121].

According to recent reports, orally administered styrene maleic acid (SMA) nanomicelles encapsulating epirubicin can pass through the intestinal epithelium without affecting its tissue integrity and exhibiting dual uptake by the enterocytes and microfold (M) cells thereby acting as a potential anti-cancer drug delivery system [122].

Ion pairing complex of oxaliplatin (OXA) with N-deoxycholyl-l-lysyl-methylester (DCK) (OXA/DCK) and 5-fluorouracil (5-FU) incorporated into water-in-oil-in-water nanoemulsions exhibited enhanced oral absorption. Oral administration of nanoemulsion containing OXA/DCK and 5-FU in the colorectal adenocarcinoma cell (CT26) bearing mouse model resulted in tumor growth inhibition by 75% in volume [123].

Zein nanocapsules have been found act as potential therapeutic carriers augmenting oral co-delivery of resveratrol and exemestane in breast cancer models [124]. On the other hand, co-delivery of doxorubicin and silybin against hepatoma is observed to be manifested by oral hepatic targeted liposomal formulations [125].

Nanomicelles composed of soluplus, D-α-tocopheryl polyethyleneglycol succinate and dequalinium have been reported to augment cellular uptake and anti-cancer effects in the drug-resistant breast cancer MCF-7/Adr cell line. The co-localization of these nanomicelles with the mitochondria activates the mitochondria dependent apoptotic pathway. Oral administration of paclitaxel loaded nanomicelles in MCF-7/Adr-xenografted BALB/c nude mice has been found to exhibit ameliorative effects [126].

Oral delivery of β-casein nanomicelles induces its targeted release in the stomach and helps in overcoming the glycoprotein dependent multi-drug resistance in gastric cancer. SN-38, a breast cancer resistance protein (BCRP) transport substrate and the BCRP efflux transport inhibitor, elacridar, exhibit high binding affinity to β-casein nanomicelles and hence, can be used as an effective drug delivery system [127].

Another study showed that poly (3-hydroxybutyrate-co-3-hydroxyvalerate) carriers not only exhibited low cytotoxicity by itself but also increased the bioactivity of 5-fluorouracil i.e., the cytotoxicity to kill HT-29 human adenocarcinoma cells [128]. The same nanocarrier has also been shown to enhance the cytotoxicity of silymarin in HT-29 cells and to penetrate 3D micro tumors leading to significant decrease in their size [129].

Studies related to the therapeutic effects of irinotecan hydrochloride or metformin hydrochloride loaded poly-lactic-co-glycolic acid nanoparticles for the treatment of glioblastoma multiforme exhibited significant reduction in the tumor volume following administration of the nano-drug complex [130].

Thus, all the studies discussed above showed that various types of oral nanomedicines are used to treat different types of cancer [[128], [129], [130], [131]].

## 9 Toxicity of oral nanocarriers

Nanoparticles exhibit a wide range of toxic effects on entering a biological system. For example, ceftriaxone loaded chitosan nanoparticles of size 210 nm and 45% drug encapsulation efficiency, exhibits cytotoxicity when incubated for 24 h at a dose of 1.8 mg/mL in Caco2 cell line [132]. In such cases, combinatorial therapy involving antioxidants is a possible solution to reduce the toxic effects of the nanocarrier. For example, taurine is reported to attenuate nano-copper induced oxidative damage of hepatocytes by modulating the mitochondria dependent and NF-κB mediated apoptotic pathway [133]. The toxicity is dependent on the physic-chemical properties, nature of the material, size, shape, surface properties and biodegradability of the orally administered polymeric nanoparticles [134]. Administration of poly-lactic-co-glycolic acid–polyethylene oxide copolymer at concentrations of 3, 15 & 75 μg cm^−2^ in TK-6 cell line, incubated for 4, 24 & 48 h respectively, induce significant increase in the number of micronucleated binucleated cells (MNBNCs) [135]. However, in most cases, oral nanocarriers reduce the toxic effect of the free drug when administered in conjugation [136]. Oral administration of poly-c-glutamic-chitosan nanoparticles, normally used for the treatment of diabetes at a dose of 100 mg kg^-1^ body weight, in normal IRS mice maintains normal values of haematological and biochemical parameters [137]. On the other hand, administration in C127I mouse breast cancer cell line at a concentration of 0–2 μg mL^-1^ has shown that the cytotoxicity of tamoxifen (Tmf) loaded nanocarrier (Tmf-PLGA) is much less than free Tmf [138].

## 10 Conclusion

Exploitation of nanotechnology for targeted drug delivery is an important therapeutic approach. Taking advantage of its size restriction at the nanoscale, nanoparticles can successfully act as carriers for important therapeutic agents. Cost effectivity and ease of application have favoured the oral route for drug administration in the developing countries. Studies related to structural modifications of these nanocarriers have helped in evading such problems. The oral administration of a wide array of nanoformulations like liposomes, dendrimers, niosomes, cubosomes, chitosan nanoparticles, nanoemulsions, nanocrystals etc. loaded with proteins or drugs have been found to be effective in ameliorating the adverse effects of different diseases. Oral nanostructures not only find their application in the field of drug delivery but also in cases of gene therapy and vaccination. The oral route augments controlled release and enhanced permeability & retention (EPR) effect of nanomedicines, thereby contributing to their increased efficacy as therapeutic agents. Extensive research is necessary for the improvement and modification of such oral nanoformulations to make those drugs more effective in their applications.

## Conflict of interest

The authors share no conflict of interest.
